# Low-cost green recyclable biomaterial for energy-dependent electrical switching and intact biofilm with antibacterial properties

**DOI:** 10.1038/s41598-020-71610-w

**Published:** 2020-09-03

**Authors:** Muhammad Sultan Irshad, Naila Arshad, Iftikhar Ahmed, Misbah Sehar Abbasi, Muhammad Idrees, Shafiq Ahmad, Mohamed Sharaf, Muhammad Sohail Asghar, Mazen Zaindin

**Affiliations:** 1 Ministry-of-Education Key Laboratory for the Green Preparation and Application of Functional Materials, School of Materials Science and Engineering, Hubei University, Wuhan, 430062 People’s Republic of China; 2 Institute of Quantum Optics and Quantum Information, School of Science, Xi’an Jiaotong University (XJTU), Xi’an, 710049 People’s Republic of China; 3 Energy Research Centre, COMSATS University Islamabad, Lahore Campus, Lahore, 54000 Pakistan; 4 School of Energy and Power Engineering, Xi’an Jiaotong University XJTU, Xi’an, 710049 People’s Republic of China; 5 Department of Physics, COMSATS University Islamabad, Lahore Campus, Lahore, 54000 Pakistan; 6 Department of Industrial Engineering, College of Engineering, King Saud University, Riyadh, Saudi Arabia; 7 Department of Physics, University of Wah, Wah Cantt, 47040 Pakistan

**Keywords:** Biochemistry, Biophysics, Chemistry, Physics

## Abstract

A highly cost-effective recycled biomaterial extracted from lime peel has been made biocompatible and has been coated on a commercial fluorine-doped tin oxide (FTO) substrate of glass using the spin coating method. Structural, morphologic, electronic, and antibacterial measurements were thoroughly characterized as a green biomaterial thin film using X-rays (XRD), PL, FTIR, Raman, SEM, HRTEM, AFM, I–V, and antibacterial diffusion techniques. The comprehensive analysis of structures of recyclable waste in the form of lime peel extract (LPE) as thin films showed the crystalline cellulose structure that corresponds to the lattice fringe (0.342 nm) exposed by HRTEM. The K^+1^ interstitial active sites or vacancies in LPE/FTO thin films are confirmed by the PL spectra that show important evidence about conduction mechanisms, and hence facilitates Ag^+1^ ion migration from the top to the bottom electrode. The AFM investigations revealed the minor surface roughness (169.61 nm) of the LPE/FTO film, which controls the current leakage that is associated with surface defects. The designed memory cell (Ag/LPE/FTO) exhibits stable, reproducible electrical switching under low operational voltage and is equipped with excellent retention over 5 × 10^3^ s. Furthermore, owing to presence of flavonoids and their superior antioxidant nature, lime peel extract powder shows tremendous antimicrobial activity against gram-positive and Gram-negative bacterial strains.

The importance of various fabrication techniques of modern process engineering and nanotechnology cannot be overlooked, particularly with the emergence of ultra-smart devices, smart tools, nano scaffolds, and molecular machines^1,2^. Intelligent devices with electronic and biomedical and antibacterial applications have facilitated scientists’ research as well as other innovators who are developing new applications for endless opportunities in modern scientific endeavours^3^. Nanomaterials exist in various geometric shapes, such as wires, rods, particles, fibers, and fullerenes that exhibit unique surface-to-volume ratios and physical and chemical properties that induce characteristics related to diverse material and matrix applications in energy, water, environmental deprivation. In addition, they show resistance to introducing even diffusion in any matrix^4^. Thus, a detailed inquiry into these properties at the nano-scale is inevitable. The literature reveals that thin film coatings offer a range of core properties that serve as the pivot for state-of-the-art technologies, for example, anti-static role, wear and tear, enhanced adhesion, resistance to destructive behavior, optical spikes, mirrors and magnetic tools, molecular-scale semiconductor devices, food technologies, etc.^4,5^.

Many studies have reported diverse applications of biomaterials from natural resources, and this topic has been the focus of attention for researchers and scientists across the globe due to their medical, biomedical, and medicinal (clinical antioxidant, antibacterial, and antitumor) applications^4,6^. Contrary to previous beliefs, the bark or peel part of fruits have appeared as more valuable components of agriculture products as compared to conventional beliefs^7^. There are a number uses for citrus fruits due to their coherence, economy, and bio-compatibility. Citrus has been used as a reservoir for nutraceutical and many dietary supplements^6,7^. The peel and pulp of the fruits have confirmed anti-toxicity and anti-pollutant properties, and it is a candidate material for future sustainable structural engineering applications^7,8^. The biomaterial of citrus peels and the derived products have been extensively employed for various energy conversion and storage system devices, such as organic solar cells (dye-sensitized solar cells, DSSCs), batteries, fuel cells, supercapacitors, and non-volatile memory devices^7–9^. Nonetheless, citrus also has sources for pectin, which has been reported as a base material for non-volatile memory microsystems that are largely used in data storage applications^5,9^.

Our research group initially reported that citrus peel fruit extract byproducts (pectin) could intelligently scavenge certain cations, such as Ca^+2^ and Zn^+2^, from the effluents released in wastewater streams^1,6^. These enormous properties are outsourced from the chemical structure and composition of the fruits, as illustrated in Fig. 1. In particular, orange and mandarin citrus have proved important sources of valuable bioactive molecules^10^. Recent research has attributed extraordinary significance to the novel nutritional and medicinal valuable sub-products present in citrus^10,11^. Citrus peels are used as a store of essential units, including soluble sugars, organic acids, carbohydrates, and carotenoid fibres^11–13^. Many of the flavonoids serve living bodies by stabilizing the free radicals participating in various human metabolism redox activities^9,11^. The yellowish-green color in vitamin C is associated with citrus lime fruit^11^.

**Figure 1:**
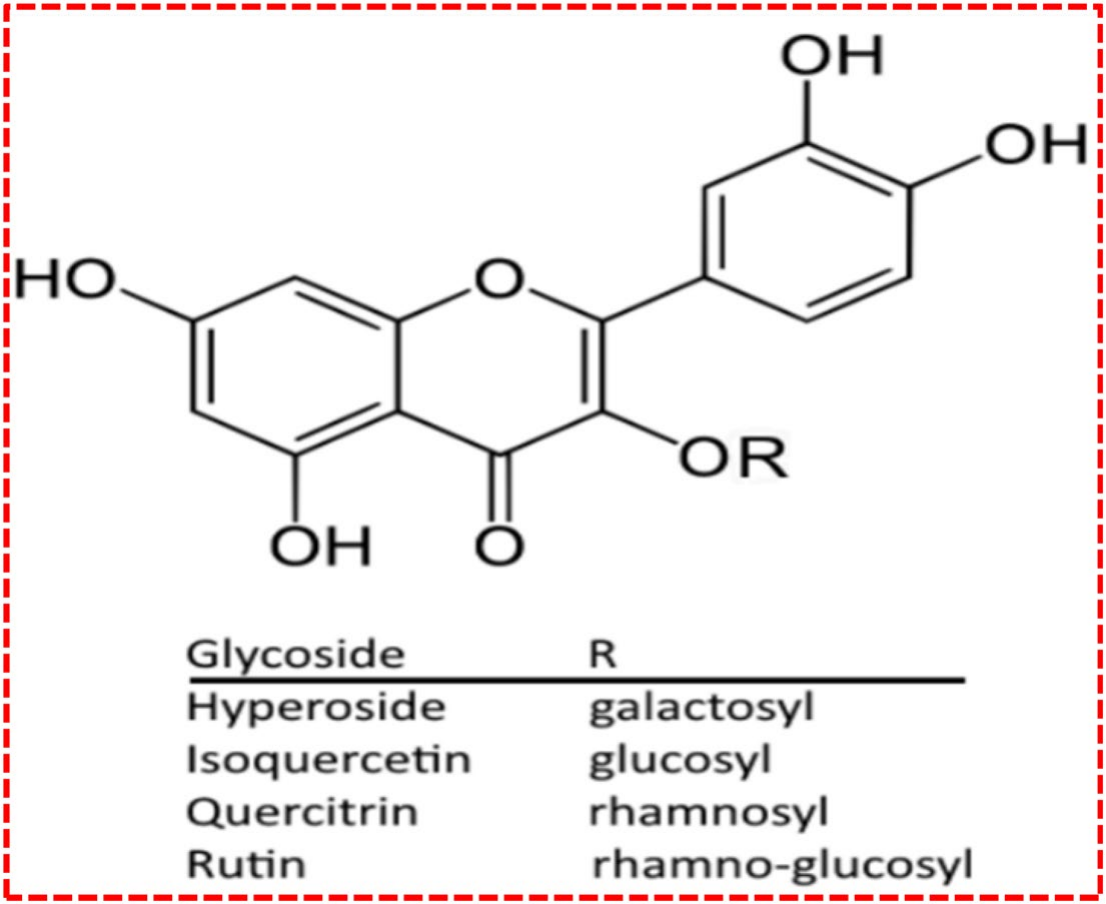
Represents the basic functional group and structure of flavonoids^13,15^.

In addition to the above uses, the good fragrance and absorbent capability have made citrus part and parcel of cosmetic products. Limonene and flavonoids are sub-products that can help physicians to control obesity, aging, carcinogenic activity including tumour growth, etc.^11,12^. Lime peel contains two unique ingredients, albedo and flavedo, in the external part of the peel that appear greenish-yellow. A flexible, songlike, cellulose film in lime peels contains albedo^14,15^. Various medical conditions can be cured or managed with the byproducts of citrus plants, including inflammation and allergies and has anti-mutagenic and carcinogenic activities. Multiple formulations of food contain flavonoids that help with prevention before a disease therapy is engaged^12,13,15^. Apart from organic medicinal ingredients, the lemon fruit peel offers a host of minerals, such as potassium (K), Sodium (Na), Calcium (Ca), Magnesium (Mg), Copper (Cu), Zinc (Zn), Phosphorus (P), and Iron (Fe)^16–18^.

Our presented work deals with a simplified laboratory fabrication of stacked thin film consisting of LPE/FTO. Moreover, the structure and composition of the films are explored for their sustained antimicrobial bioactivity as well as the resistive switching phenomenon. The presence of the flavonoid structure confirms the potent applications in healthcare-related products on account of unique content and composition^14,15^. According to recent research, appreciable work is available simultaneously for uses of biomedical germicide strips as well as recyclable, reproducible electronic switching in memory devices. This report provides another dimension to the dual applications of biofilm in medical care (antibacterial agents) and in sustainable, recyclable electrical memory devices.

## Results and discussion.

### Crystal structural analysis (XRD)

**Figure 2:**
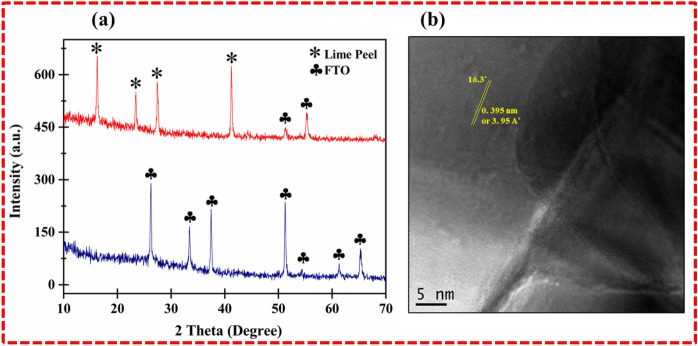
(**a**) Demonstrate the XRD crystallographic spectrum of bionic thin film of LPE/FTO that expose the cellulosic crystalline structure originate due to van der walls forces or hydrogen bonding of flavonoids molecules. (**b**) HRTEM image of lattice fringes also confirmed the strongest peak d-spacing of bionic LPE thin film on FTO substrate.

The two theta ranges, 20°–80°, are explained in Fig. 2a, which shows an X-ray diffraction study of the biofilm (lime peel extract coated on the FTO substrate). The crystalline rutile phase FTO assisted as a substrate, which imparts significant crystalline properties in the matrix compared to bare glass. Nonetheless, the LPE-based biofilm shows sharp peaks, confirming the presence of the overall crystalline phase. Fundamentally, raw lime peel extracts exhibit cellulose, lignin, hemicellulose, and flavonoids compounds with their derivatives^19–22^. The LPE-coated thin film on the FTO substrate demonstrates the crystalline structure equipped with sharp peaks of crystalline cellulosic components and few rutile FTO substrate peaks. The existence of cellulosic crystalline peaks is due to the hydrogen bonding of adjacent molecules or van der Walls forces^19^. The observed two-thetas angles of 16.3°, 21.8°, 26.6°, 41.1°, 51.9°, 55.6° correspond to crystalline cellulose and flavonoid compounds with their derivatives. Other peaks of the FTO substrate appeared to show congruence at certain points in the spectrum, e.g., 51.9°, 55.6°. The standard cellulosic crystal structure is visible at 16.3°, 21.8°, while 26.6° may find the origin in flavonoids, and 41.1° can be referred to as the rutin type of flavonoid or hesperidin. The greater alignment in the polymeric material triggered fibrous molecular level rearrangement in the LPE/FTO films, which resulted in the fibrous assembly of the lime peel stretching via physical extraction of the material. The HRTEM lattice fringe of the LPE thin films perfectly match the XRD evaluated d-spacing (0.395 nm or 395 Å), as illustrated in Fig. 2b. The crystalline size of the lime peel’s extracted material was calculated to be 118.55 nm using the well-known Scherrer's equation from Table 1.

**Table 1:** Denotes the estimation of crystallite size parameter of lime peel extract thin films deposited on FTO substrate followed by well-known Scherrer equations and brags law.

No	Angle (2̾θ)	FWMH	d (Å)	Lattice strain (%)	D (nm)
1	16.3	0.230	3.95	0.0054	38.43
2	21.8	0.134	2.43	0.0035	40.38
3	26.6	0.236	1.52	0.0042	28.47
4	41.1	0.152	2.13	0.0020	32.75
5	51.9	0.216	2.09	0.0031	36.32
6	55.6	0.113	1.47	0.0045	38.61

Average crystallite size = 41.25 nm.

Average lattice strain = 0.0065.

### Identification of functional group (FTIR and Raman)

**Figure 3:**
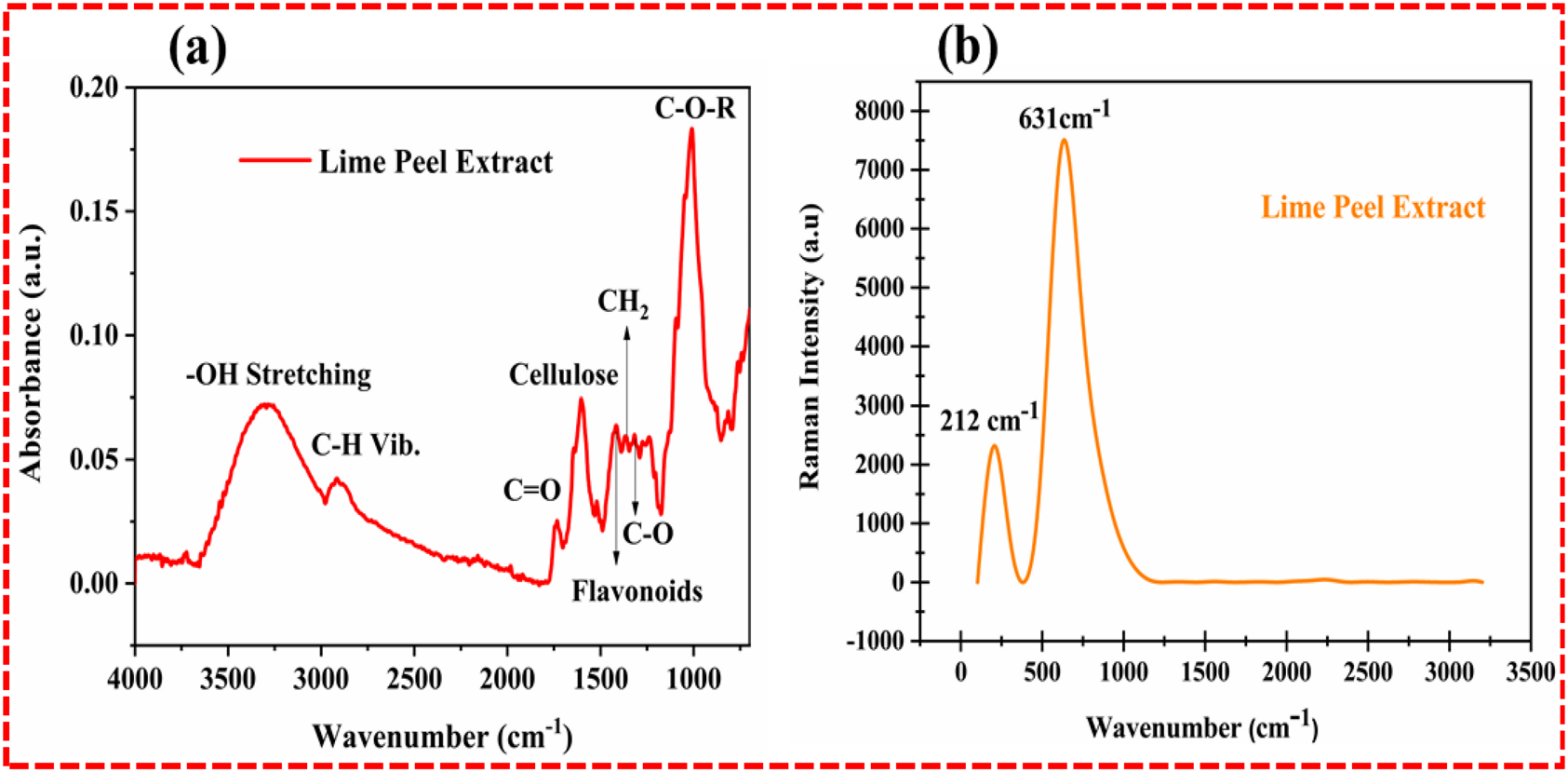
(**a**) FTIR spectrum exposed various class of flavonoids in LPE thin film that’s drive bioactivity and bioionic electrical switching as a dilectric layer. (**b**) The broad absorption in visible region for π–π^*^ transition within LPE matrix revealed by Raman spectra.

The functional groups present in the thin LPE film were explored using FTIR Spectroscopy. Figure 3a refers to the LPE extracted film range spectrum, from 500 to 4000 cm^−1^. The band of high intensity at 3415.22 cm^−1^ can be attributed to the stretching frequency of the hydroxyl (–OH) functional group present in carbohydrates. The FTIR peak at 3019.76 cm^−1^ can be referred to as hydrocarbons (C–H) groups due to either the symmetrical or asymmetrical stretching vibrations. The band at 1685.18 cm^−1^ is related to the carbonyl (C=O) functional group due to the stretching vibrations. Another intense FTIR signal at 1134.31 cm^−1^ indicates the ether-linkage (C–O–R), and the signal peak of 1385.48 cm^−1^ that refers to the near-aliphatic hydrocarbon chains (–CH_2_– and –CH_3_ ) reflects the structural basis of the material^23^. The bending vibration signal at 1591.65 cm^−1^ of C–H can be correlated to quercetin-type flavonoids. Moreover, the FTIR signal at wavenumbers 1735.67, 1488.63, and 849.15 cm^−1^^22,23^ were found to be associated with pure cellulose, alcohol, organic acid, and alkyl halide, respectively.

As shown in Fig. 3b, the LPE extract’s energy absorption was investigated by Raman spectroscopy and was evaluated in the range 0–3500 cm^−1^. Raman bands at 212 cm^−1^ and 631 cm^−1^ for a thin LPE film can be linked to carotenoids, which is a type of flavonoid^23^. The citrus fruit is rich in flavonoid carotenoids, which result in the lemon fruit’s coloring and which offers natural healthy products for consumers. Strong-colored pigments originate from the permitted π–π^*^ transition towards a visible region^24^. As soon as the excitation laser frequency is applied in coherence with the electronic shift in carotenoid molecules, a Raman spectrum^23,24^ resonance is established. Thus, the Raman spectral band intensity is enhanced, contrary to normal Raman scattering. In basic vibration modes, the probability of combinations and overtone increases. Hence, ultraviolet or visible excitement contributes to resonance acquisition.

### Defect chemistry of LPE/FTO

**Figure 4:**
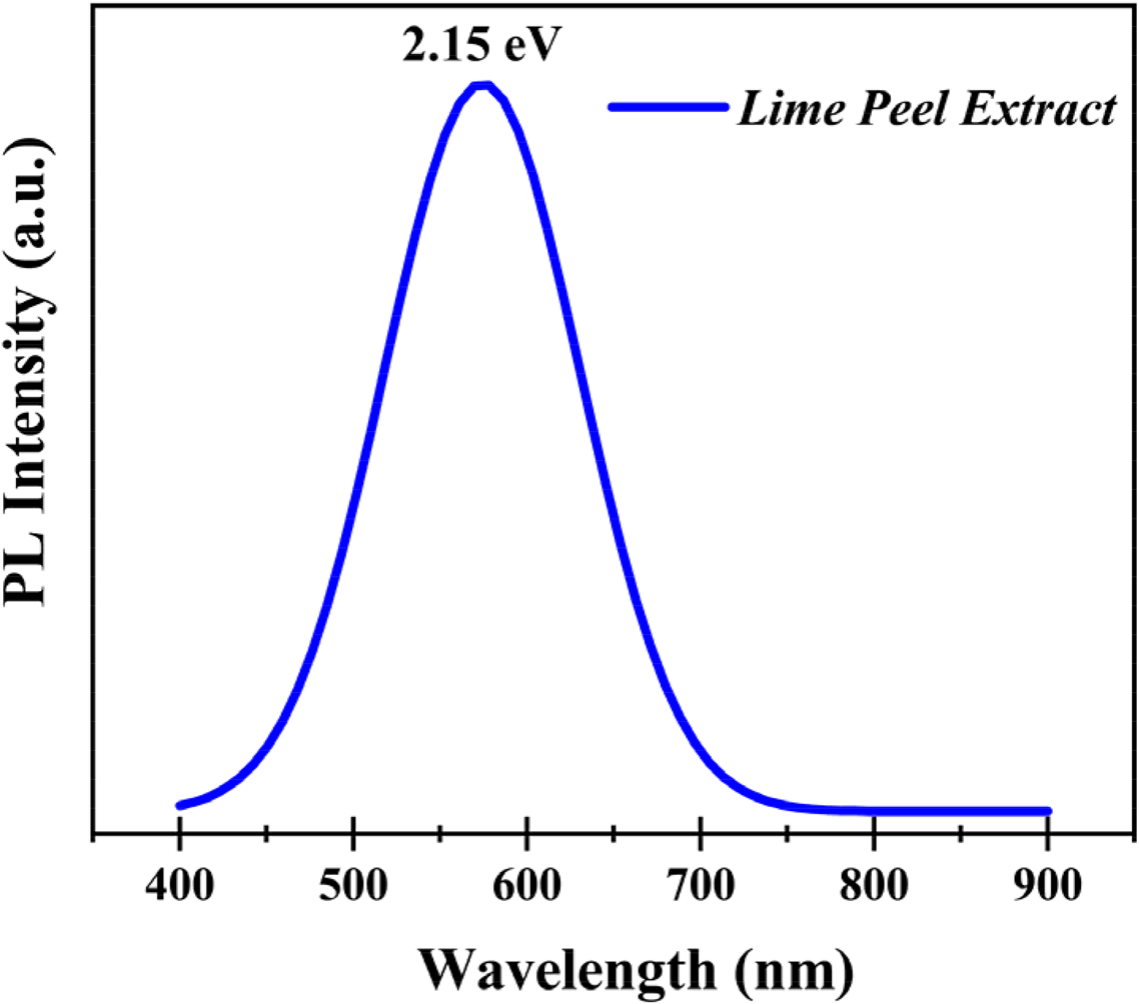
Pl spectra comprehend the defect chemistry of interstitial K^1+^ defect abundant LPE thin film that ease the diffusion of Ag^1+^ ion from the top to bottom electrode.

Photoluminescence (PL) spectroscopy can analyze the spontaneous intrinsic defects existing in biomaterial thin films and can investigate defect chemistry to explore excitation and recombination of charges in the lattice or point defects that appeared during synthesis, such as active structural and interstitial defect sites^25^. Figure 4 explains the PL spectra of thin film coated over FTO at an excitation wavelength of 457 nm and laser exposure time of 10 s. Only one sharp intensity peak was observed at 577 nm, corresponding to 2.15 eV bandgap of LPE/FTO thin film in the visible yellow region^25,26^. There exist no additional peaks in the spectrum due to the good quality of the prepared biomaterial. In fact, two types of defects are responsible for irradiative recombination, which may lead to sharp, intense peaks. Radiatively transitioned mobile electrons, if trapped in deep bulk levels near the valence band, can cause electron trapping^26^. Hence, electrons held in a trapped state are aligned with a structural defect that may be potassium ions, K^+1^ active site in structure, or vacancies. These active interstitial sites are considered to ease the migration of the Ag^+1^ ion from the top to to bottom electrode. The peaks related to the trapped electrons are those with a wavelength range of 590–700 nm that lie about 0.70–1.60 eV to the red band^24^. If we consider the hole-trap mechanism, then the mobile electrons that are in a radiative course transition can recombine with the trapped hole in a deep and shallow bulk level, which is found below the range of the conduction band. The deep shallow larger states are linked to oxygen vacancies, which originate the PL emission signal peaks in a range of colors varying from the yellow or green region with a 500–570 nm wavelength at 1.8–2.5 eV^24–26^. The LPE spectrum signal shows a recombination of mobile electrons with the holes at a deep shallow and bulk level under the conduction band via a radiative channel transition at 577 nm, referred to as a 2.15 eV bandgap.

### Morphology and surface roughness view

An additional investigation regarding surface morphology along the texture profile of the LPE/FTO films was executed using Atomic Force Microscopy (AFM). The aggregation of the surface of lime peel films can be explored more convincingly if they are compared with natural cellulose xanthate (CX) biomaterial-based thin films, which show slightly less roughness than LPE thin film^27–29^.

**Figure 5:**
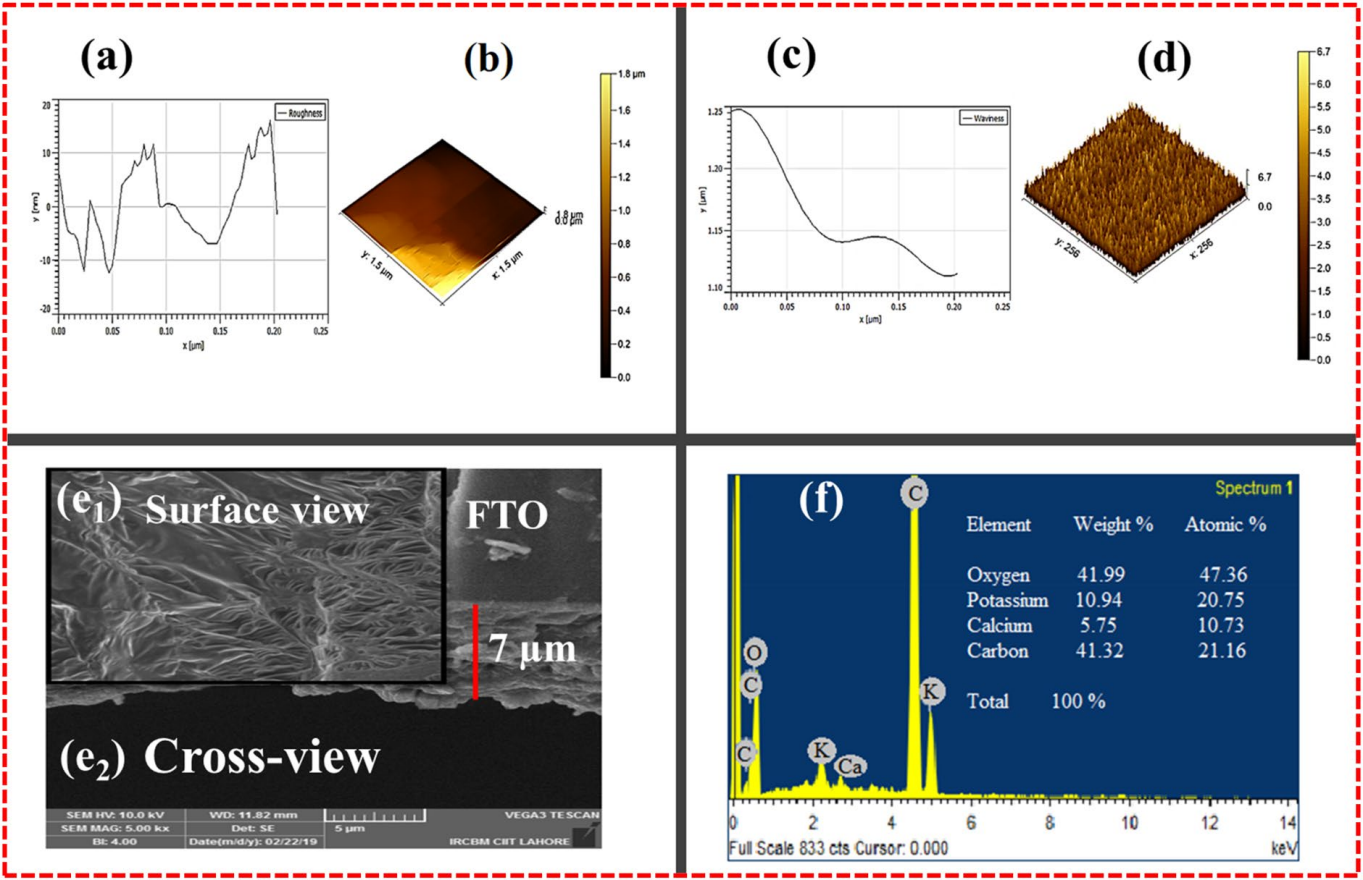
Morphology and topography of natural bionic LPE/FTO thin film. (**a**) Surface roughness spectra that shows negligible surface defects or dangling bond. (**b**) 3D AFM surface view of LPE/FTO. (**c**) The topographical waviness spectrum. (**d**) 3D AFM surface waviness. (**e1**) SEM surafce view of cellulosic crstalline homogenious growth of LPE on FTO. (**e2**) SEM cross-view exposed the LPE film thickness on FTO substrate. (**f**) Basic elemental composition of bioactive compounds present in LPE/FTO thin film via EDS.

Indeed, surface topography and the roughness of thin films decisively affects the electrical switching nature by avoiding surface defects or dangling bonds. These superfluous surface defects cause an annoying conducting channel, which may be the origin of the electrical deterioration of memory devices. As shown in Fig. 5a, the surface roughness of the LPE/FTO thin film revealed a negligible root mean square (RMS) roughness value (169.61 nm) that shows a regular pattern and homogeneous growth, as demonstrated in the AFM 3D view of surface topography equipped with a dimension of (1.5 × 1.5 µm) as shown in Fig. 5b. The statistical estimations from the well-known Gwyddion software show a median of 0.5328 µm in an under-selected area of 5.410 µm^2^. The slightest roughness appeared due to the stress of adhesion between the FTO substrate and LPE, and this adhesion pull enhances the strength and long-term stability of the deposited film. The deposited LPE thin films can be visualized in a broader context, in terms of the smooth waviness, in a 3D view of the waviness profile, as illustrated in Fig. 5c and d. The waviness spectrum demonstrates the smoothness wave shift and depicts the regularity of the pattern in a selected area of 256 × 256 µm. A brief statistical analysis estimated the average magnitude of height spacing to be 0.027, 0.053, and 1.437 µm, and the AFM investigations were compared with SEM and HRTEM microscopies. The role of surface roughness and interfacing cannot be ignored in electron scattering, as they promote the studied deposited films’ electrical conductivity. The same impact has been observed on the hole mobility of the transistor and the electron’s mobility of the reverse phase layer (inversion part) of the film matrix. The observed surface morphology of the LPE/FTO thin films, as shown in Fig. 5e1, shows regular cellulosic deposition, and its adhesion factor shows minor stiffness on the surface, which is also observed in the AFM 3D view of surface topography. The surface view of the LPE/FTO thin films also exposes the smooth growth of cellulosic crystalline structures, which confirms the XRD findings and the HRTEM lattice fringes observations.

Henceforth, it is concluded that a regular patterned layer of LPE is associated with an electrical resistive switching signal due to a lack of surface porosity to manage the deterioration of the switching layer. Additionally, the surface smoothness plays a significant role, and the thickness of the deposited films also matters. In order to observe the thickness of the LPE deposition on FTO substrate, a cross-view analysis was also carried out. Figure 5e2 shows the SEM cross-view, the deposited LPE with an approximate thickness of 7 µm on the FTO substrate, and the cloudy part of the cross-view reveals the cellulosic fiber of the LPE. Furthermore, an EDS analysis was also done to comprehend the elemental composition of the LPE that revealed the organic and bionic nature owing to its carbon, calcium, potassium, and oxygen components, as illustrated in Fig. 5f.

### I–V characteristics of Ag/LPE /FTO RRAM

The world is keeping a record of every passing moment, which requires a robust and dynamic data storage system. The data storage mimics the neuroscience of the human memory record system. Thus, resistive random-access memory (RRAM) has now acquired a level of maturity that needs to be made more viable with low energy consumption, long memory retention, facile fabrication, rapid on–off switching speed, and non-volatility^9,20,29,30^. A variety of biomaterials have been already tested for fabrication flexibility, electronic swing, sustainability, economy, elasticity, and organic nature^30^.

Organic semiconductors attracted the researcher’s attention due to its outstanding optoelectronic properties^30^. Many biomaterials drawn from fruits have been investigated for resistive switching memory devices^30,31^. The resistive switching devices operate by varying the resistance through a dielectric medium present as a sandwich between two metallic electrodes^31,32^. The dielectric materials possess the unique property of storing charges at the interface of electric polarization under an applied voltage. The phenomenon of resistive switching can be identified by two types of applied polarity, known as unipolar and a bipolar^30–33^. Bipolar conduction behavior is also termed as asymmetric and can be underlined by the current–voltage (I–V) curve, which helps determine the existence and development of a conduction filament operating through a dielectric biomaterial thinly configured over a substrate such as FTO. The electroforming step requires a range of positive voltage (0–5 V) over a top layer electrode with a step voltage range of 0.1 V and a current compliance value of 10 mA, keeping safety measures to avoid permanent breakdown of the dielectric media^31–33^, while the FTO substrate glass is kept grounded. In the case of the LPE thin layer as the dielectric media, it is switched suddenly to the least resistance state (LRS) at 1.5 V, which affirms the electroforming phenomena that may be varied and optimized under electric stimulation.

**Figure 6:**
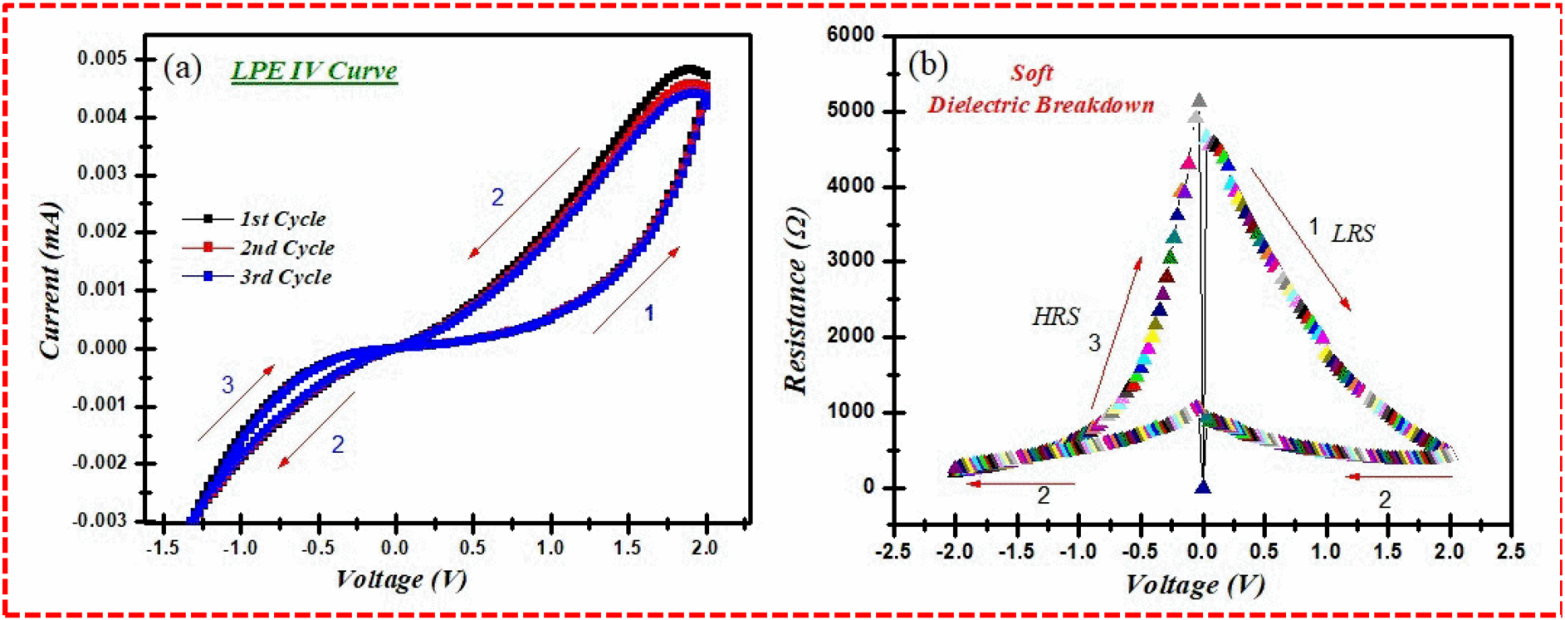
(**a**) Represent the bipolar electrical switching curve of bionic LPE/FTO RRAM cell. (**b**) The electrical switching purely depends threshold voltage dependent resistance switching at room temperature.

Figure 6a exhibits the full curve of the LPE resistive switching at a voltage range of the order of 0 V → 2 V → − 2 V → 0 V under room temperature conditions and 5 mA current value. As recently reported by Wang et al.^17^, a low current range is inevitable to maintain safe protection from a permanent dielectric breakdown of the biomaterial-based device. An operational voltage ranges between 0 to 2 V (sweep1). The LPE as the dielectric media offers a linear output of up to 1.4 V, and when suddenly depressed to a low resistance state (LRS) with a rise in current from 1 to 50 mA, the set state is defined as the ON state. When the sweep follows at a voltage range of 2 V → − 2 V, the LPE dielectric media maintains an LRS. Nonetheless, under inverted conditions and a reverse-phase at 2 V → 0 V, the LPE media is sustained in an LRS up to − 1.3 V and jumps abruptly to a high resistance state (HRS), which is termed as the OFF state, at a voltage range of − 1.4 V, which further ratifies the asymmetric or bipolar resistive switching phenomenon exhibited by the LPE dielectric media. The process was repeated for three successive cycles, which confirmed the existence of both resistive states (LRS/HRS). This behavior is conceivable and may be reproduced at a voltage range of 1.5 V/− 1.4 V without any significant reduction. Figure 6b shows the resistive variable profile of the LPE media under study at a full cycle sweep of 0 V → 2 V → − 2 V → 0 V. Initially, in the first run, the resistance changed suddenly to 200 Ω to 5 kΩ at 1.5 V, which shows LRS. The most attractive feature observed in the second run at 2 V → − 2 V is that the resistance profile remains consistent without any changes to the LPE media at approximately 200 Ω. In the third run (3rd sweep), the LRS switched again and turned suddenly to HRS at − 1.4 V. This variable resistance at a voltage range ratified the resistive switching behavior of the LPE biomaterial device under study.

**Figure 7:**
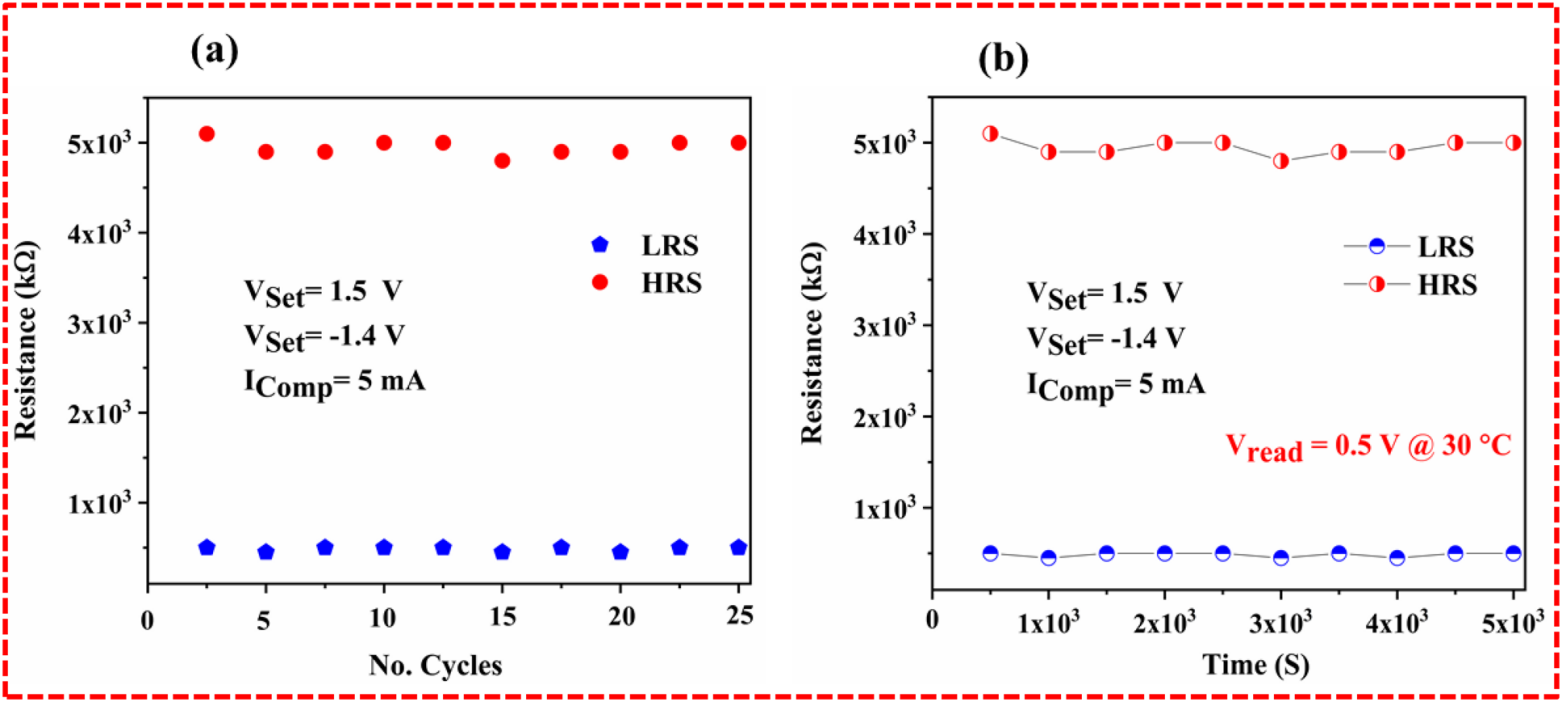
(**a**) Endurance profile of bionic Ag/LPE/FTO RRAM for 25 consecutive cycles. (**b**) Excellent retentivity over 5000 s along reproducible bipolar switching nature.

The ON/OFF ratio is the prime characteristic of RRAM that reveals the resistance variation window. This resistance variation window attributes the stable set/reset state or writing and erasing data of the resistive switching memory devices. Normally, organic materials are less mature than inorganic materials owing to their thermal and chemical stability. A fabricated memory device has better stability at room temperature along with low error detection under a minimum voltage supply. The ON/OFF ratio was examined to understand the efficient resistive switching across different temperatures, as illustrated in Fig. 7a. It was found that the maximum ON/OFF ratio (4 × 10^3^) was observed at 30 °C under 0.5 reading voltage. The limitations of reading the voltage lies in the chemical instability of the lime peel extract. The obtained ON/OFF ratio replicates the efficient resistance window that differentiates the set and reset state of the memory device. The maximum efficiency of the ON/OFF ratio of the LPE RRAM designates the chemical stability of host material, which is gradually decreased under a successive applied thermal response. Moreover, the statistical mean values of the distinctive voltage were evaluated to analyze the voltage distribution probability of the fabricated Ag/LPE/FTO-based RRAM. As shown in Fig. 7b, the set/ON state and reset state/OFF state at 1.5 V/− 1.4 V was confirmed through statistical voltage distribution (Tables 2 and 3).

**Table 2:** Represent the functional groups present in lime peel extract and the observed stretching vibrations modes estimated from FTIR and Raman spectroscopic investigations of lime peel extract based thin films.

Wave number (cm^−1^)	Bond	Functional group
849.15	C–Cl	Alky halides
1134.31	C–O–R	Ether linkage
1385.48	–CH_2_– and –CH_3_	Aliphatic chains
1488.63	C–O	Alcohols, carboxylic acid, ester
1591.65	C–H	Flavonoid
1685.18	C=O	Carbonyl
1735.67		Cellulose
3019.76	C–H	Alkanes
3415.22	–OH	Carbohydrates

**Table 3:** All the assays of lime peel extract powder against carcinogenic pathogens were repeated in triplicate and their mean values $\pm$ standard deviations and represent their inhibition zone profile.

LPE concentrations	Zone of inhibition (mm)			
	Gram positive		Gram negative	
	*E. coli*	*Klebsiella pneumonia*	*Bacillus subtilis*	*S. aureus*
20 ug/mL	14 ± 2	6 ± 2	9 ± 2	14 ± 1
50 ug/mL	19 ± 1	11 ± 1	13 ± 1	16 ± 2
80 ug/mL	22 ± 1	15 ± 2	15 ± 1	18 ± 1
DMSO	11 ± 1	0	8 ± 1	0
Ciprofloxacin	29 ± 0	28 ± 2	30 ± 1	11 ± 2

**Figure 8:**
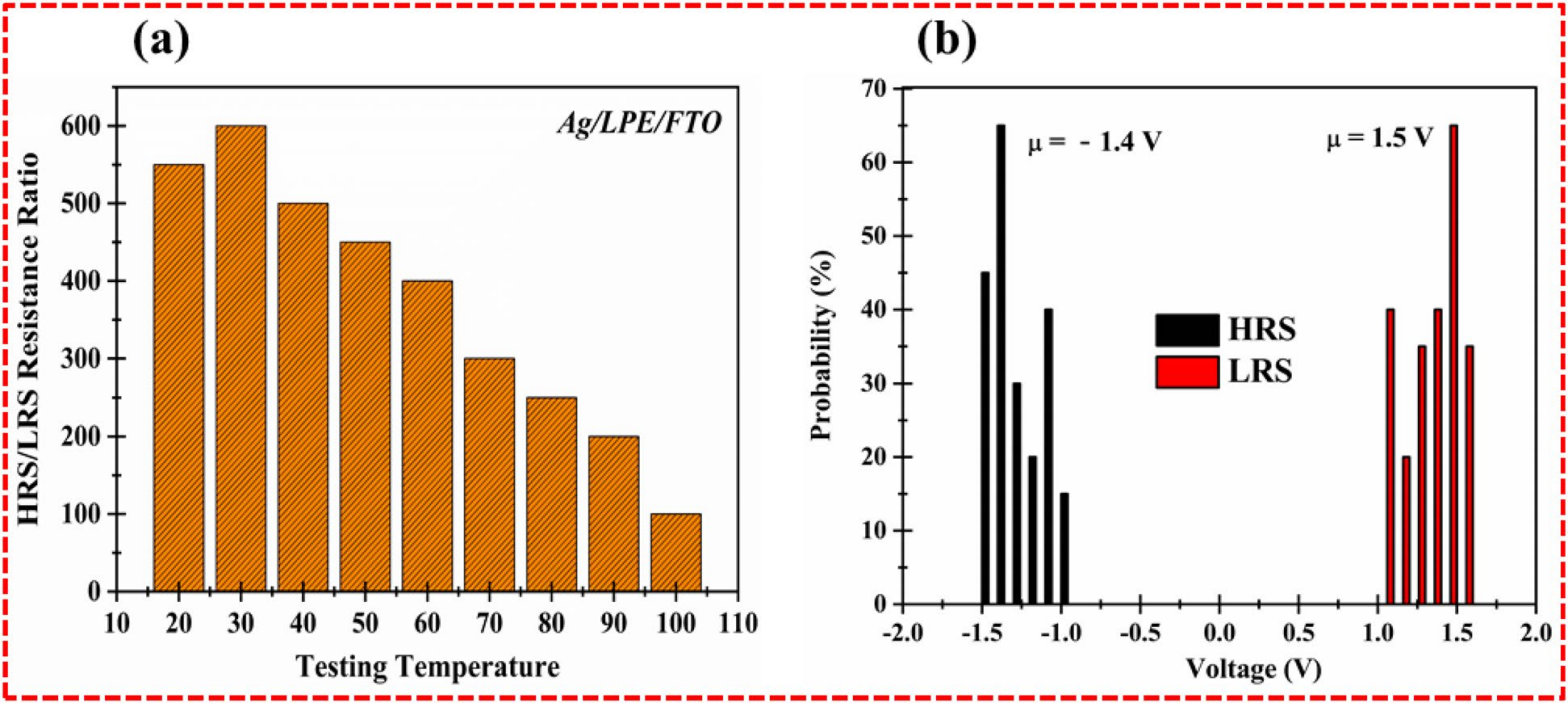
(**a**) ON/OFF ratio of Ag/LPE/FTO RRAM profile on various tesiting temperature. (**b**) Stable and reproducible voltage-dependent electrical switching was confirmed by statistical voltage distribution evaluation.

The least possible electrical degradation plays an essential role in electrical switching and promotes the longevity and viability of memory devices^29–31^. Tremendous efforts have been made to avoid electrical degradation by introducing new materials that offer better electrical switching stability^32^. In this contribution, the low cost and eco-friendly lime peel extract-based RRAM devices demonstrate excellent endurance (4 × 10^3^) under a successive 25 cycle-to-cycle variation because of its better resistance variation stability, as depicted in Fig. 8a. The electrical switching stability over a 25 cycle-to-cycle variation and its excellent resistance window make Ag/LPE/FTO RRAM a better option for use in green wearable flexible electrical switching devices. As shown in Fig. 8b, a retention test was carried out to observe the electrical degradation of the LPE-based RRAM over 5 × 10^3^ s. Certainly, the fabricated memory cell exhibits a smaller attenuation factor after 5000 s, which shows the excellent retention of the Ag/LPE/FTO-based RRAM. The observed performance parameters increase the interest in low-cost biomaterial utilization in memory storage devices.

**Figure 9:**
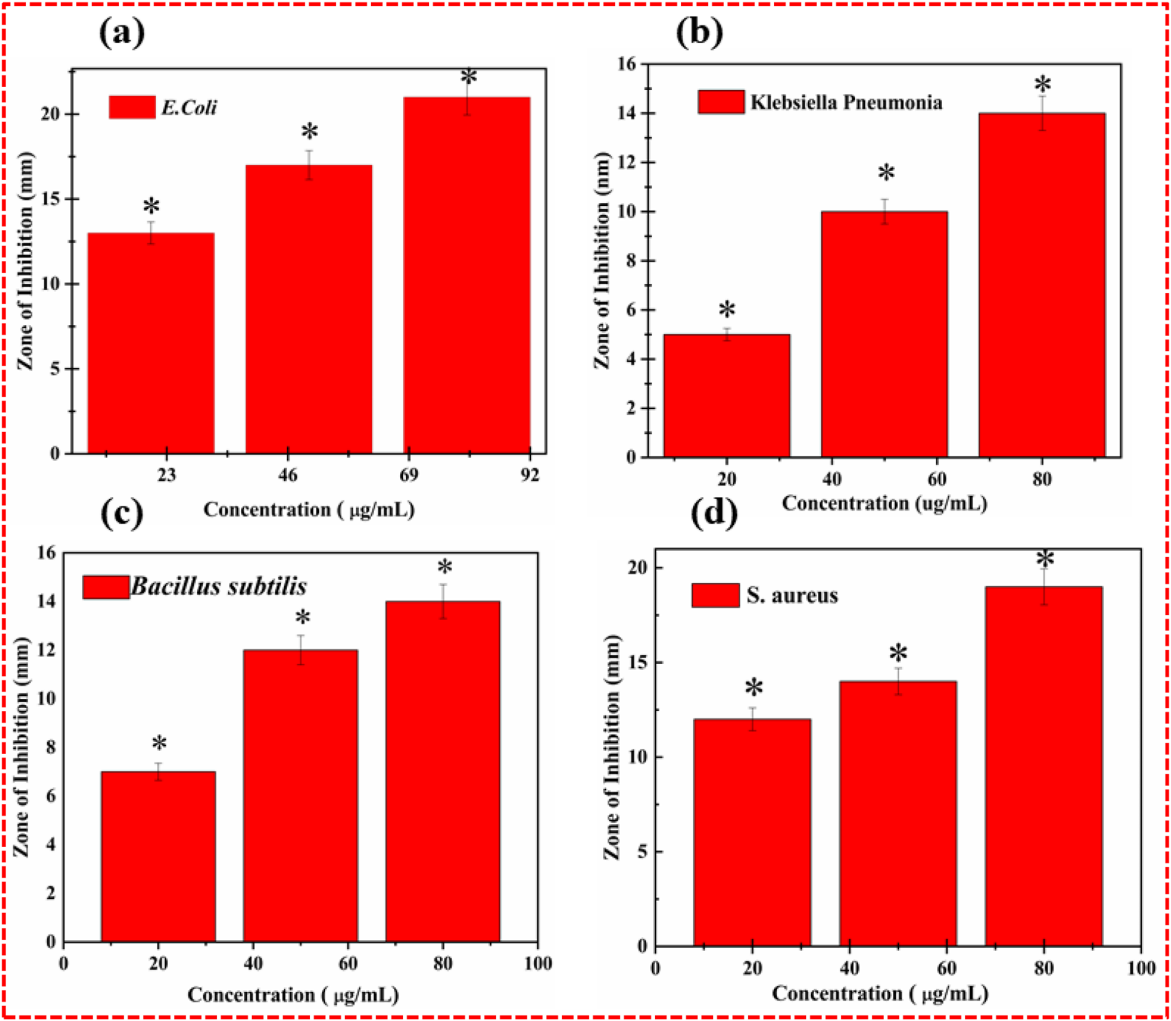
Flavonoids rich LPE powders exhibits excellent antibacterial response in terms of wide inhibitory findings against various bacterial pathogens (**a**) *E. coli* (**b**) *Klebsiella pneumonia* and negative control (**c**) *Bacillus subtilis* (**d**) *S. aureus*.

Various concentrations of lime peel extract (LPE) essays were selected to investigate the antibacterial response of LPE powder against four various bacterial strains (both gram-positive and gram-negative), followed by the agar well diffusion technique. The concentrations were taken at 20 mg/mL, 40 mg/mL and 80 mg/mL. The monitored in vitro investigation was performed to bring familiarity with lime peel extricates NPs to those who use them to treat different infections brought about by subsequent contagious plagues. These pathogens were disengaged and distinguished by the analysts of the Pakistan Council of Scientific and Industrial Research Complex and Laboratories Lahore. The information found from the in-vitro investigations of lime extricate powder exposes an antioxidant nature that promotes doze-dependent treatment. The following antimicrobial analysis consisted of dimethyl Sulfur oxide (DMSO) as a solvent, and ciprofloxacin as the antimicrobial control. The centralization of anti-infection agents was 0.005 mg/mL, while the volume of lime peel extricates was 100 uL. The centralizations of the lime strip powder were 20 mg/mL, 50 mg/mL, and 80 mg/mL. As per the LPE, the NPs fixations expanded, and the respective antibacterial inhibition zone likewise expanded because of its cell reinforcement flora of flavonoids, as in Fig. 9.

## Discussion

**Figure 10:**
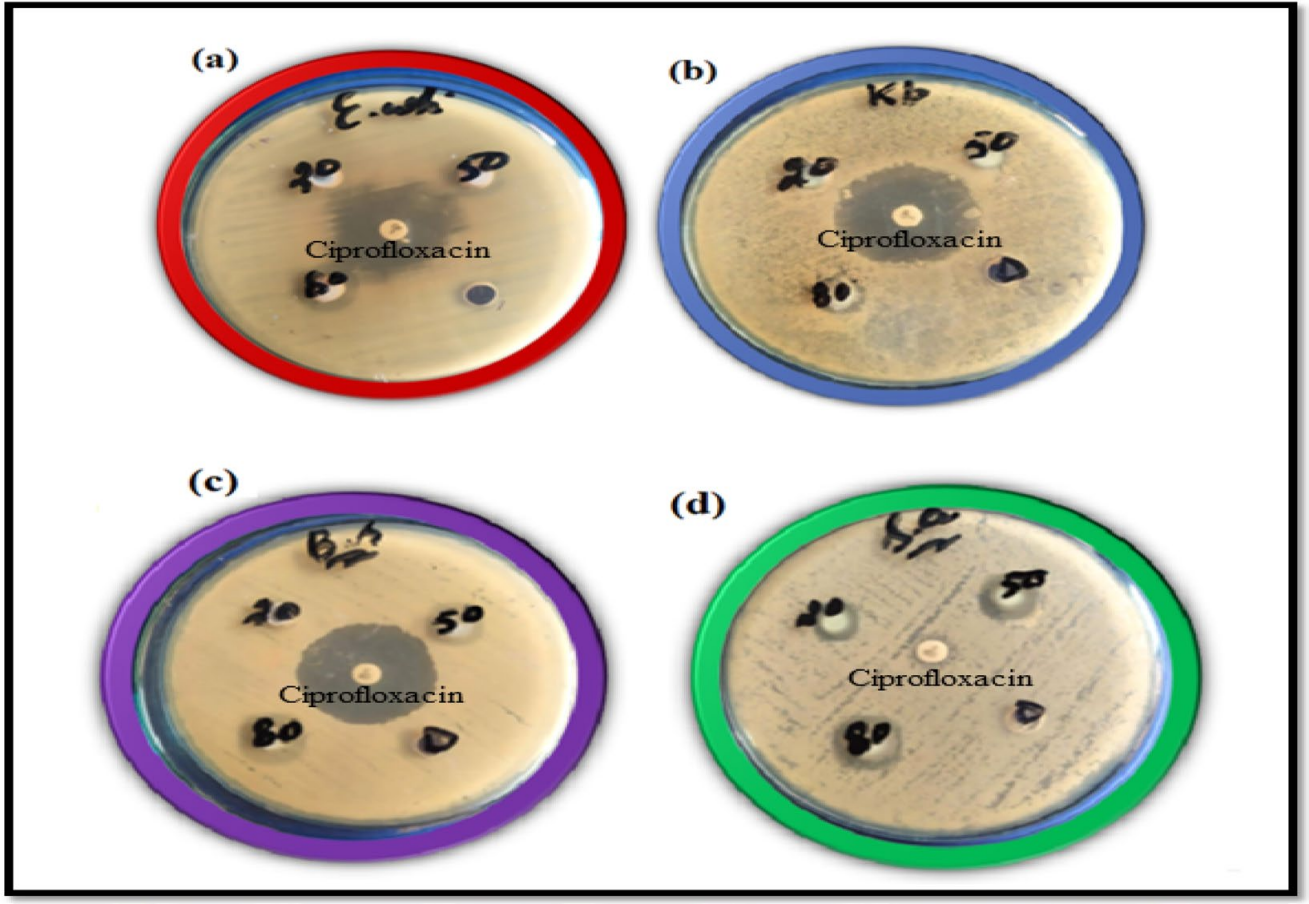
Antimicrobial activity of lime peel extract against different microbial strains at different concentrations with accordance to positive and negative control. (**a**) *E. coli* (**b**) *Klebsiella pneumonia* (**c**) *Bacillus subtilis* (**d**) *S. aureus*.

The citrus peel has two sections: flavedo and albedo. In the external colored part, the flavedo is present. Flavedo is very small and holds the oil glands along a natural wax layer^34^. It has a cellular structure, and the inner area exhibits multipart intercellular structures that consist of the albedo part, which is porous and white (gas volume) sponges^34^. The flavedo’s natural waxes were considered to avoid water losses from the surface of the plate33,34. In comparison with fresh samples of color, the dried citrus peel and leaves showed numerous variations^35,36^. Color is an indicator of the quality of vegetables and citrus fruits, whereas the rinse is based on the observer's appearance and pragmatic conditions^36^. The dramatic color variations have a significant effect happening in the organoleptic characters of dehydrated citrus peel constituents and can simply reduce or improve the application of these materials^37^. The aeration temperature and airflow proportion had no substantial effects on redness, lightness, or yellowness in the electrical measurements (Fig. 10).

Past studies have shown that conductivity depends on fruit acidity, and there is a higher average electrical conductivity in acid fruits than in sweet and sub-acidic fruits. Lemon is naturally acidic, whereas its pH is somewhat less acidic and has a pH between 2.2 and 3.0 with a mixture of vitamins and other nutrition additives for human consumption^38,39^. The citric acid in the lemon juice is primarily 5–8%, which is used as a pile and induces chemical properties^40,41^. Overall, lemons generate the most voltage due to their high acidity level^40,41^. To test their conductivity to store energy, a cutting-edge driver was inserted to generate voltage as a battery designed to incorporate three varieties of lemon fruits. Lemon is composed of voltaic cells that convert chemical energy to electrical energy. Therefore, the total power generation for a wide range of applications is very small due to a lower acidity concentration in the lemon. However, it may be used in small scales, for example, in light-emitting diode (LED) lighting^42,43^. Subsequently, conductivity depends on temperature and the accurate electric conductivity of an intact lime peel. When fragmented, the lime peel has difficulty conducting some temperatures^39^. Intact tissue is not altered due to thermal disruption from the change in electrical conductance, when it is altered by an increase in sample temperature^39^. In other words, the electrical conductivity of the elementary tissue was not affected considerably through the material temperature as compared to the completely disintegrated tissue^39–41^ of the lipid bile layer and the incorporated protein from the cell membrane. A major function of the cell membrane is to control the passage of substances into and out of cells. The cell membrane's decay results in a reduction in the concentration of a high-conductive fluid, which is usually trapped within intact cells^41–43^. The dissemination of the electrolytes after the tonoplast to the cytoplasm improved the complete tissue conversion. With a slightly higher temperature around 30 °C, deprived of the presence of a cell membrane, more migration of electrolytes is possible from one electrode to another, leading to a considerably higher electrical conductivity^41–44^. Therefore, the viscosity, pH, and electricity of citrus juices are not altered^43–45^. There was no change in the absorption spectra of bionic LPE. Furthermore, the composition of organic acid and flavor compounds do not change significantly in color^40,41,43,45^.

**Figure 11:**
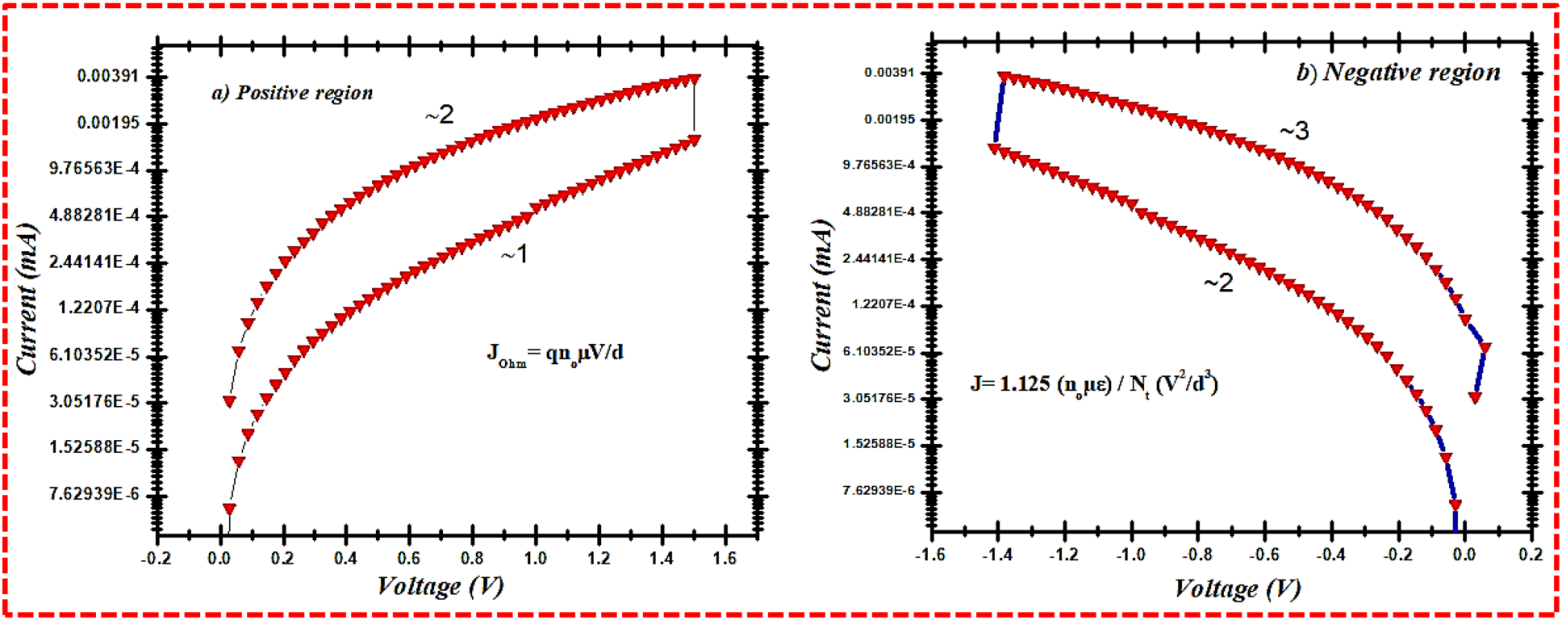
Electrical switching states experimentally verified by corresponding conduction model. (**a**) LRS followed ohmic conduction model during positive region. (**b**) HRS obeyed Child’s square law during negative region.

To comprehend the conduction mechanism of Ag/LPE/FTO-based RRAM, both the positive and negative curves of resistive switching are examined through the logarithmic, linear fitting plot. From the linear fitted graph (Fig. 11a), the initial voltage sweeps from 0 to 1.5 V and replicates the LRS switching followed by ohmic conductance when the linear fit approaches 1. Equation (1) demonstrates the ohmic conduction relation.

1 $$ J_{Ohm} = qn_{o} \mu \frac{V}{d} $$

where J_ohm_ shows current densities, q means an electronic charge, n_o_ is the charge density symbol, μ means charge mobility, and d is the active layer thickness measurement.

A classic SCCL model can enhance the conductive activities, bringing a cutting edge to the HRS that designates the conductive belt underneath the trap that can form the defect sites in the lime peel (as illustrated in Fig. 11b), and the load carriers being injected can be kept^9,46^. At the same time, pectin, carbonyl compounds, and amino acid bonds in the lime peel could have a chemical composition in an electrophilic location, where the simultaneous transmission fulfils the child square law^9,46^:

2 $$ J_{Child} = \frac{9}{8}\frac{{n_{o} \varepsilon \mu }}{Nt} \left( {\frac{{V^{2} }}{{d^{3} }}} \right) $$

**Figure 12:**
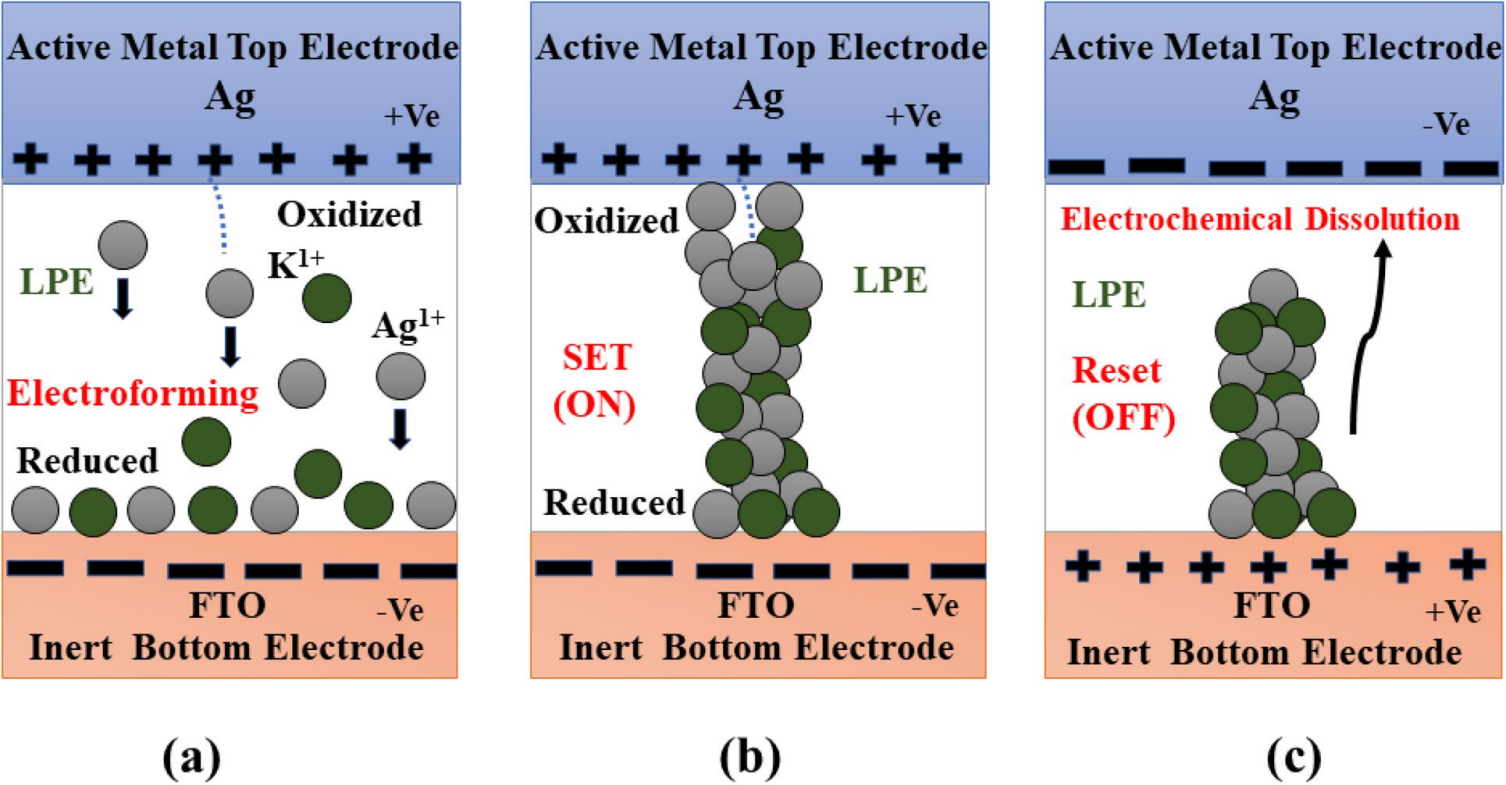
The complete comprehension of diffusion of Ag^1+^ ion assembled with K^1+^ ion within LPE matrix from top electrode to bottom electrode. (**a**) Electroforming phenomena carried out by applying positive voltage on top active Ag electrode and FTO remain grounded. (**b**) Set state or ON state achieved by electrochemical metallization by the reduction of assembly of Ag^1+^ and K^1+^ ions through electrostatic attraction pull. (**c**) reset state or OFF state switched by electrochemical dissolution at top electrode interface by applying opposite polarity.

The resistive switching phenomena of fabricated Ag/LPE/FTO-based RRAM followed the well-known conduction mechanism "Electrochemical Metallization (ECM)" due to the incorporation of an active Ag metal top electrode. As shown in Fig. 12, the schematic illustration of the migration of a highly active K^1+^ ion combined with an oxidized Ag^1+^ ion. Owing to its highly electropositive nature of a silver oxidizer under a positive voltage sweep at the top interface into highly conductive positive ions (Ag^1+^) that trigger potassium into a K^1+^ ion as well within the LPE matrix. The followed oxidations of both silver and potassium are Ag → Ag^+1^ + e^−1^ and K → K^+1^ + e^−1^, respectively. The same type of oxidation of a potassium ion has been observed in a recently reported orange peel-based RRAM^17^. The migration of the oxidized Ag^1+^ and the K^1+^ ion is driven by the electrostatic pulling of negative polarity at the bottom electrode or is followed by an electric field that can be visualized in Fig. 12a (forming process). The LPE thin-film matrix facilitates the driving of these active positive ions through their lattice defects of potassium vacancies. The successive load of the positive sweep across the top electrode and due to highly electropositive character enabled Ag/LPE/FTO RRAM to establish a conductive bridge between the top and bottom electrodes by reducing the Ag^1+^ and K^1+^ ions. This conductive bridge or filament gave birth to conduction or a short-circuit between a sandwich structure (top/bottom electrode) and led towards an LRS, or ON state, as depicted in Fig. 12b (set process). The obtained LRS was achieved at a specific voltage (1.4 V) and termed as a set voltage or threshold voltage. After attaining LRS or a soft dielectric breakdown, the memory cell stayed in LRS until the applied polarity altered. Due to its bipolar resistive switching nature, a conduction filament dissolutes at the top Ag/LPE interface under a negative sweep following an electric field direction, as illustrated in Fig. 12c. This electrochemical dissolution corresponds to a reset state or OFF state of the Ag/LPE/FTO-based RRAM under opposite polarities. Both states are reproducible without any attenuation or electrical degradation.

**Figure 13:**
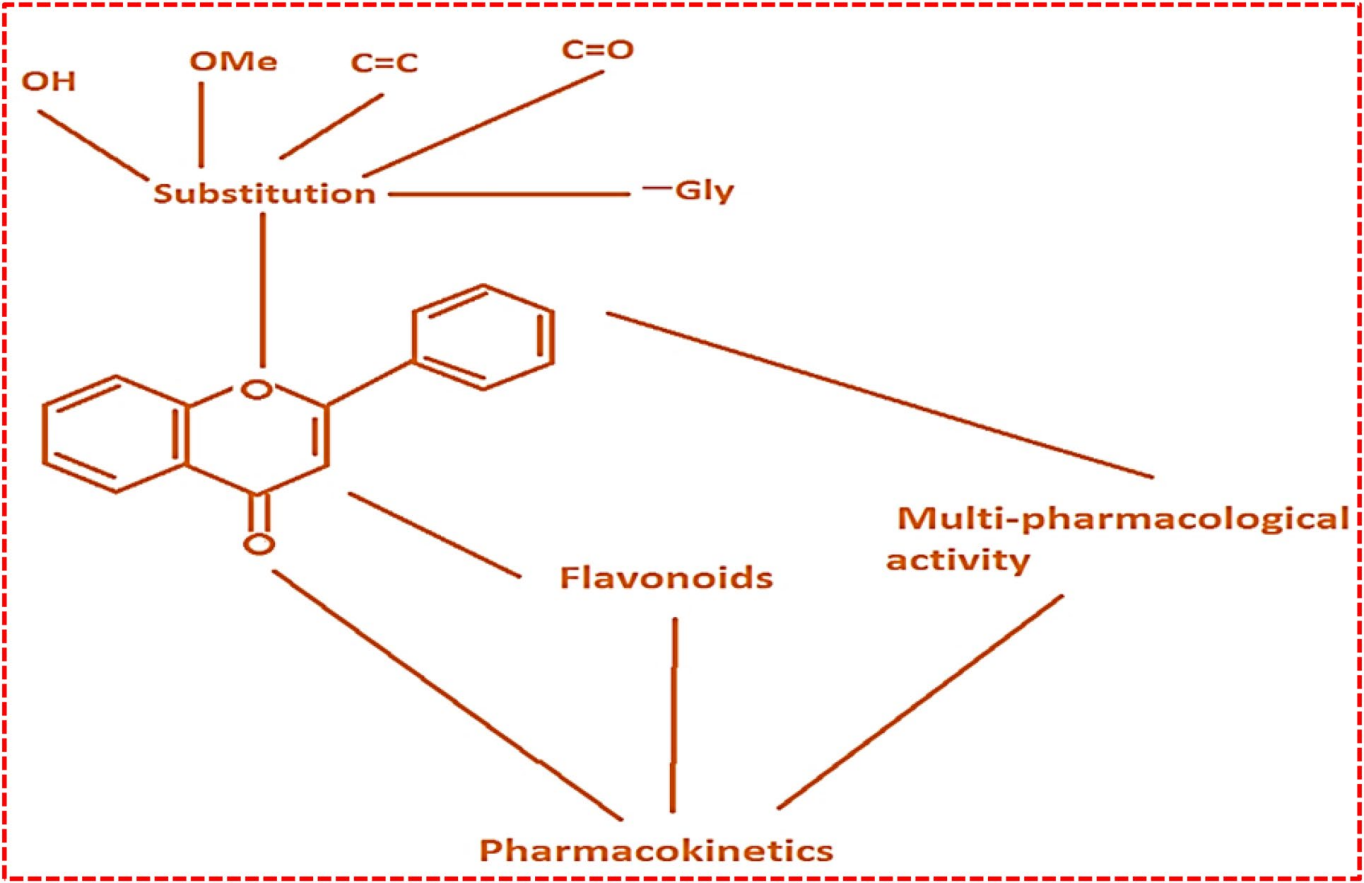
Unique chemistry of three carbon linkage with respected two phenyl rings enabled it to destruct the cell wall membrane of bacterial pathogens^50^.

However, our team reported that the peel extract could be used as a selective trapper to remove cations from wastewater, as previous studies have said that the bioactive and bionic compounds that exist in natural citrus fruits that are rich with fiber, folic acid, flavonoids, limonin, vitamin C, and β-carotene effectively reduce the risk of progressive ailments^8,11,47–49^. These precious flavonoids compounds are connected to a family of compounds of plant origins that can be used effectively and that have the effect of inhibiting microbial virulence, synergy through antibiotics, and straight antibacterial activity^46–49^. Basically, these flavonoids are polyphenolic complexes, which are mainly are known for their antiviral, cancer-fighting, antibacterial properties, their impact on capillary fragility, and their tendency to block the antagonistic effects of human platelets^46,49^. Despite the high loss rate of flavonoids and flavanols in natural citrus fruits associated with respective flavanones, they play an important role in antibacterial activity^47,48,50^. For each flavonoid, the attainment of antioxidant nature leads a combination of 2,3-double bond and 4-oxygenation functions. Two hydroxy groups are positioned at three and five along with the unique O-dihydroxy assembly in B-. There is a major category of polyphenolic complexes with a strong C6–C3–C6 backbone^47,48,50,51^. As shown in Fig. 13, the two benzene rings are coupled through a small chain of three carbon linkage bonds^50,51^.

The unique feature of the lime peel is its three carbon chains that trigger oxidation and saturation of the chemical stability of flavonoids. Different subgroups of flavonoids have been identified in higher plants. To date, more than 8000 various classes of flavonoids compounds have been identified^7,9,50,51^. Poly methoxy flavonoid aglycone, glycoside (naringin) and flavonoid glycoside (rutin) are the species of flavonoids present in citrus fruits. Their presence has been found in plants and used to tackle infectious pathogens, cancerous cells, and fungi in a defensive role^47,50^. The antibacterial properties associated with the structure of flavonoids are linked primarily due to its structural chemistry that exhibits aromatic rings along with hydrophobic swaps, as illustrated in Fig. 13, i.e. phenyl. The excellent ability of flavonoids molecules to block different bacterial virulence influences quorum detection in the form of enzymes, signal receptors, and toxins, respectively^51–53^. Thy also provide favorable results for *S. aureus* alpha-toxin poisoning and Helicobacter pylori infection. A true description and portrayal of the antibacterial activity of gram-positive and gram-negative bacteria and five different strains may indicate the presence of broad-spectrum antibiotic compounds in the extract. The chosen microorganisms for analysis were gram positive *S. aureus* and gram-negative *E. coli*. The antibacterial activity of dried citrus fruits was assessed. Previous research has identified that deviations in the cell wall structure may cause an excellent antibacterial response in Gram-negative bacterial pathogens as compared to Gram-positive. The thickness of the cell wall plays a decisive role in the antibacterial response in Gram-negative bacteria, especially *E. coli*, which has a 7–8 nm cell thick wall that is composed of lipopolysaccharide and peptidoglycan. In contrast, the depth of Gram-positive Staphylococcus aureus ranges from 20 to 85 nm, which is a relatively thick membrane (cell wall) comprised of many mucins, lipoproteins, and Mureic acid^54,55^. Also, Staphylococcus aureus contains antioxidant enzymes and has strong antioxidant properties. Flavonoids, especially the choroid, can prevent certain Gram-positive and Gram-negative bacteria that cause many normal and deadly random diseases^54–57^. These ailments comprise folliculitis, skin-care infections, cellulite, skin burning syndrome, swelling, blood infections, meningitis, hepatitis C, toxic shock, and sepsis^53–58^. Recent developments have revealed the positive therapeutic effects of flavonoids on various distempers, influenza viruses, etc. Flavonoids are also effective anti-cancer treatments, including orientation of apoptosis, inhibition of nuclear factor signaling, inhibition of proteasome differentiation, induction of receptor interactions, inhibition of cycle arrest, and interactions with enzymes related to carcinogenesis^56–60^. Additionally, flavonoids have precise cytotoxicity to cancerous cells, thus providing a means to discover new methods of studying inhibitors of cell growth based on flavonoids as anti-cancer drugs^55–60^.

## Method

Initially, fresh lime fruit was collected from the well-renowned Sargodha garden of Pakistan. The harvested lime peel fruit was peeled off, and their wet peel washed many times to remove the fertilizer and other compounds remove completely. Then, the cleaned peel was severed diagonally into many pieces and immersed into a deionized water/acetone solution for half an hour in an ultrasonic bath. The ultrasonic cleaning removes the impurities from inside the pores of the lime peel. Later, the collected lime peel pieces were dried in a convection oven at a certain temperature (50 °C) but avoiding complete dehydration. The dried ones were ground using a mortar and pestle, and after getting a fine powder, it was also ball milled for 2 h. The obtained powder dissolved thoroughly in alcohol for the next procedure (vacuum filtration). The extractives of the lime peel were attained through a suction filtration pump on wattman filter paper. The collected residue was dried and stored as powder for the antibacterial test. The process was repeated, and the collected powder was dispersed evenly in methanol for the fabrication of the LPE/FTO thin film.

**Figure 14:**
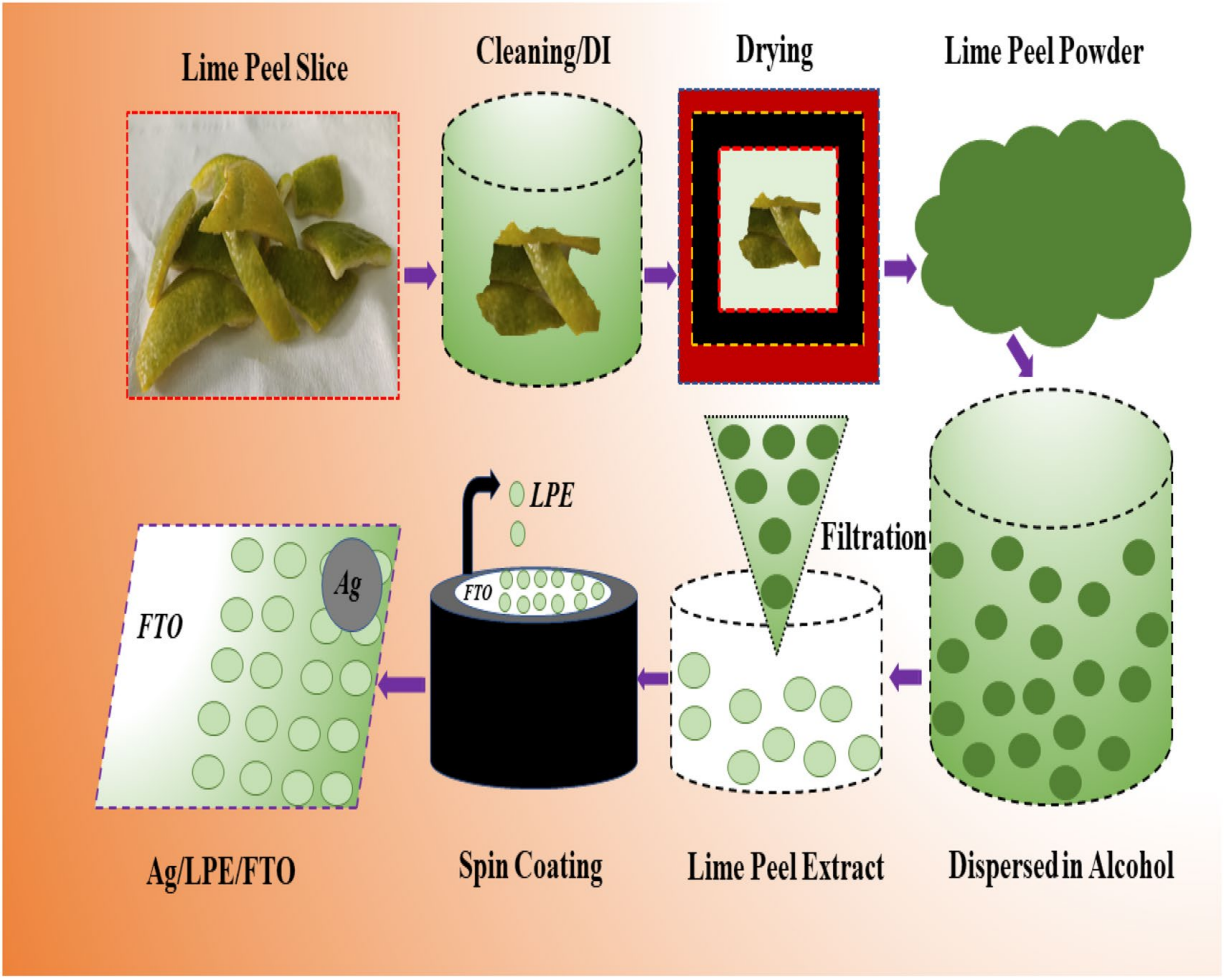
Represents the schematic diagram of complete synthesis and testing procedure of LPE films.

The fabrication of low-cost, organic bionic material lime peel extract (LPE) was processed using an easy-to-handle and well-known spin coating method. The commercially available FTO glass (1 cm × 1 cm) was cleaned ultrasonically several times and used as the substrate for the deposition of thin films. One fourth of the FTO glass was covered by sublimation tape, and the slurry of lime extractives were injected dropwise on the spinning FTO glass at 1000 RPM. Afterwords, the deposited substrate was heat-treated at 50 °C. One fourth of the FTO glass acts as the bottom electrode. For the top electrode, we used a conductive silver paste with a brush to insert a circular layer of Ag from a specified area (10.32 mm^2^) and cured it in a convection oven at 60 °C for 1 h. Herein, a complete simplisticlly designed Ag/LPE/FTO was ready for electrical resistive switching, which comprises lime peel extractives as the switching layer and silver paste and FTO as the active top and inert bottom electrodes, respectively, for memory storage applications. To understand and visualize the full experimental process of synthesizing lime peel extract, a diagram is presented in Fig. 14.

XRD (Philips PANalytical Xpert) was used to study the crystal structure information of the LPE film with Cu K alpha radiation (λ = 1.5406 Å) ranging from 20° to 70°. The surface morphology and the thickness of the film were analyzed by SEM. A Keithley 2450 (SMU) equipped with two probe stations was used to test the resistance change curve of the LPE film. An InVia Renishaw, Raman magnifying glass that exhibits an excitation wavelength of 457 nm was used for Raman spectroscopy and photoluminescence spectroscopy to analyze the defect chemistry in the LPE layer.

Four strains of bacteria, *Klebsiella pneumonia*, *E. coli* (Gram-negative), Staphylococcus aureus, and *Bacillus subtilis* (Gram-positive), were chosen from the current research projects of the FBRC, PCSIR Lahore.

We dissolved 20 µg/mL of the lime extractives in DMSO sonicate for one hour to make the blend uniform and stored it in a sterile, properly labelled, microcentrifuge tube.

To test the antibacterial response of the bionic and flavonoids in rich lime peel extractives against prescribed bacterial pathogens (Staphylococcus aureus, Bacillus cereus, *E. coli*, *Klebsiella pneumonia*), we isolated these important pathogens from diabetic foot ulcers. The standard Mueller Hinton (MHA) agar medium was used to determine the antibacterial activity using the well diffusion method. The support was prepared according to the requirements. Emulsify enough test organisms in saline to achieve absorption (equivalent to 0.5 standard McFarland). Immerse antiseptic cotton gauze in the culture solution and remove the excess culture solution by shaking the wall of the tube. Use three methods to wipe the surface of the plate with the labelled stub. Rotate the plate 60° amongst each wipe and let it parch for three to five minutes to eradicate additional moisture from the surface. Then add the microbial dilutions to the wells to check for antibacterial activity. A sterile hole bur and 100 µL dilution of lime extractives and a similar quantity of the DMSO as per the controller were dispersed to the pored well. We incubated the plate in an incubator at 37 °C for 18–24 h and recorded the diameter of the inhibitory zone around the hole and measured in millimeters. The dispersed development was recorded, the plates were incubated for an additional 18–24 h, and the diameter of the area was recorded on the second incubation day. If ambiguous findings were seen, then the experiment was repeated three times, and the average results of the three trials were verified.

## Conclusion

A spin-coated bionic and bioactive LPE thin film was fabricated on a commercially available crystalline rutile phase FTO substrate. Various characterizations, including FTIR and Raman spectroscopy, identified a variety of biological and electroactive components, such as hesperidin, quercetin, carotenoids and rutin flavonoids. An XRD analysis confirmed the crystalline cellulosic structure that perfectly matches with HRTEM lattice fringes width (0.395 nm). The photoluminescence spectrum of the LPE film revealed the presence of point defects, such as K^+1^ vacancy or interstitial active sites. These defective active sites can promote the migration of Ag^+1^ ions from the top electrode to the bottom electrode. Raman scattering located a superior class of flavonoids: carotenoids are highly colored pigments because they allow π–π^*^ transitions in the visible region. Despite the fibrous cellulose, the surface morphology of the LPE film will still aggregate and have a horizontal pattern, and no surface wrinkle irregularities were observed. The SEM results found in favour of AFM investigations. The DC resistance measurement results of the Ag/LPE/FTO RRAM demonstrated a low operational voltage-based bipolar electrical switching behavior (SET/Reset, 1.5 V/− 1.4 V), which was accompanied by a very low compliance current of 5 mA. Both LRS and HRS states can be reproduced in 25 consecutive switching cycles (e.g. 5 × 10^3^ s) without any deterioration in electrical performance. Nonetheless, the lime peel extract demonstrated significant antibacterial activity against four different strains of 20 μg/mL, 50 μg/mL, 80 μg/mL, namely *E. coli*, *Klebsiella pneumonia*, *Bacillus subtilis*, and active Staphylococcus aureus. These biomaterials with dual properties were ratified with unique characterization.
